# The Paradox Association between Smoking and Blood Pressure among Half Million Chinese People

**DOI:** 10.3390/ijerph17082824

**Published:** 2020-04-20

**Authors:** Mengying Wang, Wenyong Li, Ren Zhou, Siyue Wang, Hongchen Zheng, Jin Jiang, Shengfeng Wang, Canqing Yu, Wenjing Gao, Jun Lv, Tao Wu, Weihua Cao, Yonghua Hu, Liming Li, John S. Ji

**Affiliations:** 1 School of Public Health, Peking University, Beijing 100191, China; mywang@bjmu.edu.cn (M.W.); lwy_edu@163.com (W.L.); zhouren1994@163.com (R.Z.); yzmagiw@163.com (S.W.); zhc_1995@163.com (H.Z.); jinjiang2002@gmail.com (J.J.); shengfeng1984@126.com (S.W.); yucanqing@pku.edu.cn (C.Y.); pkuepigwj@126.com (W.G.); epi.lvjun@vip.163.com (J.L.); caoweihua60@163.com (W.C.); yhhu@bjmu.edu.cn (Y.H.); lmlee@vip.163.com (L.L.); 2 Environmental Research Center, Duke Kunshan University, Kunshan 215316, China; john.ji@duke.edu; 3 Nicholas School of the Environment, Duke University, Durham, NC 27708, USA

**Keywords:** blood pressure, tobacco smoking, alcohol consumption, interaction

## Abstract

*Background*: The association between smoking and blood pressure (BP) has been explored extensively, yet the results remain inconclusive. Using real-world evidence of a large Chinese population, we examine the effect of smoking on BP levels. *Methods*: We utilize half a million adults from the China Kadoorie Biobank (CKB) study with baseline sampling collected between 2004 and 2008. Multivariable linear regression analyses are used to estimate linear regression coefficients of smoking for systolic blood pressure (SBP) and diastolic blood pressure (DBP). *Results:* 459,815 participants (180,236 males and 279,579 females) are included in the analysis. Regular smoking is significantly associated with lower SBP (−0.57 mm Hg, *p* < 0.001) and DBP (−0.35 mm Hg, *p* < 0.001) when compared with non-smoking in men. Additionally, SBP and DBP decrease significantly among all groups of different smoking status in women (*p* < 0.001). Additionally, pack-years show negative associations with SBP and DBP in both men and women. Further analysis shows the interaction of smoking and alcohol consumption is associated with an increase of SBP and DBP (men: 2.38 mm Hg and 0.89 mm Hg; women: 5.21 mm Hg and 2.62 mm Hg) among co-regular smokers and regular drinkers when compared with regular smokers who are not exposed to alcohol consumption. *Conclusions:* A negative association between smoking and BP is observed. However, the interaction between smoking and alcohol consumption is associated with BP increase. The findings suggest the importance of considering smoking and alcohol consumption in BP control in addition to antihypertensive treatment in clinical and public health practice.

## 1. Introduction

Hypertension is the foremost precursor to cardiovascular diseases (CVDs), which accounts for 51% of deaths due to stroke and 45% of deaths due to heart disease a year [1,2]. Regarding China, the incidence of hypertension has increased from 2.9 per 100 person-years in 1991–1997 to 5.3 per 100 person-years in 2004–2009, while the percentage of hypertensive patients with controlled blood pressure (BP) remained unchanged at around 5–7% [3,4]. Additionally, 750,000 deaths were due to uncontrolled hypertension in China in 2010 [4]. 

Smoking is a global public health intervention priority [5]. Recently, several studies have been conducted to explore the effect of smoking on BP, yet the results were inconsistent [6,7,8,9]. It has been reported smoking causes an increase in BP levels and contributes to the risk of hypertension [6,7]. However, a number of epidemiological studies have found BP levels among smokers were lower than those of non-smokers [8,9]. Additionally, quitting smoking is not recommended by current clinical guidelines for the prevention of hypertension [10,11].

The association between smoking and BP may be influenced by various confounding factors [12]. Smoking and alcohol consumption behaviors, for example, often occur concurrently [13,14]. However, most of the previous studies have focused on evaluating the independent effect of smoking on BP levels, but little is known about the role of alcohol consumption in the association between smoking and BP levels. Additionally, to our knowledge, previous studies evaluating the association between smoking and BP levels had limited sample sizes in China [15,16]. During our study, we aim to assess the association between smoking and BP levels as well as examine the potential interaction between smoking and alcohol consumption influencing BP levels among Chinese populations using the data from half a million adults from the China Kadoorie Biobank (CKB) Study.

## 2. Materials and Methods

### 2.1. Study Design and Participants

A total of 512,891 participants aged 30–79 years were recruited in the baseline survey from 10 geographically diverse regions (5 urban and 5 rural) in China between June 2004 and July 2008. The study regions were chosen to ensure geographic and socioeconomic diversity, quality of disease registries, population stability, and capacity within the study areas. The CKB study design and survey methods were described elsewhere [17,18,19]. The study was approved by the Ethical Review Committee of the Chinese Center for Disease Control and Prevention (005/2004, Beijing, China) and the Oxford Tropical Research Ethics Committee, University of Oxford (UK 025-04, UK), and all participants provided written informed consent. 

The present analysis included the participants who had a systolic blood pressure (SBP) range of 70–220 mm Hg, a diastolic blood pressure (DBP) of 45–130 mm Hg, a height of 140–200 cm (males) and 130–180 cm (females), a weight of 30–120 kg (males) and 30–100 kg (females), and a body mass index (BMI) between 15.0 and 45.0 kg/m^2^. Participants with previous physician-diagnosed heart disease, stroke/transient ischemic attack, cancer, or chronic obstructive pulmonary disease, and those who stopped smoking due to physical illness were excluded. All told, 459,815 participants were included in the analysis. 

### 2.2. Anthropometric Measurements

Physical measurements included weight, height, and blood pressure. Blood pressure was measured twice by trained staff members using a UA-779 digital sphygmomanometer. When the difference between the two measurements was in excess of 10 mm Hg, a third measurement was taken and the mean of the last two measurements was recorded. The mean of the two recorded measurements was used in the analyses. Prevalent hypertension was defined as SBP ≥140 mm Hg, DBP ≥90 mm Hg, self-reported antihypertensive medication treatment, or a self-reported diagnosis of hypertension by physicians at baseline [4] BMI was calculated as weight in kilograms divided by the square of height in meters and classified into four groups of underweight (BMI < 18.5 kg/m^2^), normal-weight (BMI = 18.5–23.9 kg/m^2^), overweight (BMI = 24.0–27.9 kg/m^2^), and obese (BMI ≥ 28.0 kg/m^2^) [20].

### 2.3. Assessment of Smoking, Alcohol Consumption, and Other Covariates

Trained staff members collected detailed information on socioeconomic characteristics, lifestyle factors and medical history of the participants through a laptop-based questionnaire.

During the baseline questionnaire, questions about tobacco smoking included how often the participants had smoked tobacco at the survey time. Regarding those who did not smoke or smoked occasionally, they were asked about prior smoking history, and whether they had smoked tobacco on most days or daily. During the current analysis, participants were divided into four groups: (1) nonsmokers were defined as those who did not smoke currently and had not smoked more than 100 cigarettes during his/her lifetime; (2) ex-smokers as those who did not smoke currently or only smoked occasionally but had smoked on most days or daily in the past; (3) occasional smokers as those who did not smoke currently but had smoked occasionally in the past or had smoked at least 100 cigarettes during his/her lifetime or those who currently smoked occasionally and had not smoked on most days or daily in the past; and (4) regular smokers as those who currently smoked daily or on most days [20]. We also collected information on duration (years) of smoking and packs smoked per day for regular smokers. Regular smokers were classified into three groups of light smokers (≤10 cigarettes), medium smokers (11–20 cigarettes), and heavy smokers (≥21 cigarettes) according to packs smoked per day. Additionally, the cumulative burden of smoking was measured by pack-years.

To assess alcohol consumption, information on how often the participants had drunk alcohol during the previous 12 months was collected and, for those who had not drunk weekly, they were asked if there was at least a year prior to that when they had drunk alcohol at least once a week. Participants were divided into four groups: (1) non-drinkers were defined as those who had never, or almost never, drunk alcohol in the past year and had not drunk weekly in the past; (2) ex-drinkers were those who had drunk weekly in the past but had never or almost never drunk alcohol or had drunk alcohol occasionally, during certain seasons, or monthly but less than weekly in the past year; (3) occasional drinkers as who had drunk alcohol occasionally, during certain seasons, or monthly but less than weekly in the past year, and had not drunk weekly in the past; and (4) regular drinkers as those who usually drank at least once a week in the past year [20]. Information on type and amount of alcohol consumption was obtained for participants with regular alcohol consumption. We calculated standard drinks per day for participants (12 g ethanol per drink) [21] and divided them into three groups: light drinkers (≤2 drinks), medium drinkers (3–5 drinks), and heavy drinkers (≥6 drinks).

Self-reported total physical activity including work, commuting, housework, and recreation activities were quantified as metabolic equivalent hours per week (MET-hours/wk.). Education was classified into five levels: no formal school (illiterate), primary school, middle school, high school, college and above.

### 2.4. Statistical Analyses

Regarding hypertensive patients, we adjusted for antihypertensive therapy effects by adding 15 mm Hg and 10 mm Hg to SBP and DBP, respectively [22]. One-way analysis of variance and a Chi-square test were used to test the differences among four groups of smoking status for continuous variables and categorical variables. Multivariable linear regression analyses were used to estimate linear regression coefficients of smoking for SBP and DBP, adjusted for age, education level, alcohol consumption, total physical activity (MET-hours/wk.), and BMI. Tests for interaction were carried out with multiple linear regression analyses by setting dummy variables of smoking × alcohol consumption interaction terms in the model. We adopted variance inflation factor (VIF) to assess the collinearity between smoking and alcohol consumption.

All statistical analyses were performed using Stata Statistical Software (version 13.1, StataCorp, College Station, TX, USA). 

## 3. Results

### 3.1. Characteristics of the Study Population

Regarding the total 459,815 subjects (180,236 males and 279,579 females), 294,715 (64.09%) were non-smokers and 120,448 (26.19%) were regular smokers. There was a statistically significant difference in age structures among four groups of smoking status (*p* < 0.001). The percentage of overweight and obese was the lowest (36.03%) among regular smokers. Also, ex-smokers were more educated and drank less when compared with regular smokers. The prevalence of hypertension was 33.09% among the participants, and the rate among regular smokers was higher than that of non-smokers but lower than that of ex-smokers (Table 1).

The characteristics of the study populations by alcohol consumption status are shown in Supplementary Table S1. Concerning the participants, 212,444 (46.20%) were non-drinkers and 67,914 (14.77%) were regular drinkers. Regular drinkers were mainly males, more likely to smoke, and with higher physical activity levels compared with non-drinkers. The prevalence of hypertension in occasional drinkers was lower but that of regular drinkers was higher compared with non-drinkers.

### 3.2. The Association between Smoking and Blood Pressure

Linear regression coefficients for SBP and DBP, according to baseline smoking status by gender groups, adjusting for age, education level, alcohol consumption, BMI, and total physical activity are shown in Table 2. We found regular smoking was significantly associated with lower SBP (−0.57 mm Hg, *p* < 0.001) and DBP (−0.35 mm Hg, *p* < 0.001) when compared with non-smoking in men. Moreover, ex-smoking was associated with higher DBP (0.23 mm Hg, *p* < 0.05) when compared to non-smoking in men. Additionally, ex-smoking, occasional smoking, and regular smoking were significantly associated with lower SBP and DBP in women (*p* < 0.001). To assess the association between smoking and BP levels quantitatively, we conduced analyses based on cigarettes per day and cumulative smoking amount (pack-years). The results showed that heavy smoking was associated with lower SBP (−1.07 mm Hg, *p* < 0.001) and DBP (−0.50 mm Hg, *p* < 0.001) in men. Regarding women, smoking was associated with SBP and DBP reductions across all groups of different smoking amounts per day (Table 3). Additionally, pack-years showed negative associations with SBP and DBP in both men and women (Table 3).

### 3.3. Interaction between Smoking and Alcohol Consumption on Blood Pressure

The association between alcohol consumption and BP levels is shown in Supplementary Table S2. We found that occasional drinking was associated with a BP decrease, while ex-drinking and regular drinking were associated with increased BP levels compared with non-drinking in males. However, occasional drinking and regular drinking were associated with reductions in BP levels when compared with non-drinking in females. Blood pressure levels according to smoking and alcohol consumption status are shown in Supplementary Tables S3 and S4. 

The interactions between smoking and alcohol consumption on BP levels are shown in Table 4 and Table 5. Regarding men, 9007 participants (4.50%) were both non-smokers and non-drinkers, and 45,474 participants (25.23%) were both regular smokers and regular drinkers. There were 172,783 (61.80%) non-smokers and non-drinkers and 865 (0.31%) regular smokers and regular drinkers in women. SBP increased significantly among regular smokers and occasional drinkers (0.98 mm Hg, *p* < 0.01 for interaction), and regular smokers and regular drinkers (2.38 mm Hg, *p <* 0.001 for interaction) when compared with regular smokers without alcohol consumption in men. Additionally, the interaction between regular smoking and regular drinking was significantly associated with a higher DBP in men (0.89 mm Hg, *p* < 0.001 for interaction) (Table 4). Regarding women, there was significant interaction on a SBP increase between regular smoking and ex-drinking (3.30 mm Hg, *p* = 0.03 for interaction), regular smoking and occasional drinking (1.75 mm Hg, *p* < 0.01 for interaction), as well as regular smoking and regular drinking (5.21 mm Hg, *p* < 0.001 for interaction). Moreover, a significant interaction effect on a DBP increase was observed between regular smoking and all groups of alcohol consumption in women (all *p* < 0.05 for interaction) (Table 5).

We also conducted an interaction analysis among male participants with both regular smoking and regular alcohol consumption to assess the interaction of smoking and alcohol consumption on BP levels quantitatively. We found the interaction was broadly significant among groups with different amounts of regular smoking and regular alcohol consumption. The interaction between heavy smoking and light drinking, medium drinking, and heavy drinking was associated with 3.06 mm Hg (95% CI: 2.04–4.09), 1.99 mm Hg (95% CI: 0.93–3.05), and 2.84 mm Hg (95% CI: 0.94–4.74) increases in SBP, respectively (Table 6). 

## 4. Discussion

During this large national study in China of 459,815 participants with a wide age range of 30–79 years, we found regular smoking was associated with SBP and DBP decreases in men and women. Additionally, BP levels were negatively associated with the number of pack-years of smoking. However, the interaction effect between regular smoking and alcohol consumption on SBP and DBP increases was statistically significant in men and women. The findings address the current evidence gaps about the effect of smoking on BP levels and demonstrate the importance of considering interaction between smoking and alcohol consumption on BP levels in addition to antihypertensive treatment, suggesting important clinical and public health practice implications.

Although a number of studies have showed smoking is associated with various cardiovascular events and can act synergistically with high BP to cause a decrease in left ventricular function, no consensus had been reached regarding the association between tobacco smoking and BP levels [6,7,8,9]. Several studies have reported current smokers may have a lower BP than non-smokers in both hypertensive patients and normotensive subjects [8,9]. We also observed regular smoking was associated with lower SBP among both male and female participants when compared with non-smokers. Further, the quantitative analysis showed BP decreased more as the smoking amount per day increased and pack-years were negatively associated with BP levels. Additionally, we observed that the negative association between smoking and BP was more prevalent in females than in males. Furthermore, we saw ex-smoking was associated with a higher BP in men but a lower BP in women. Previous studies also showed smoking cessation may result in BP increases in men [15,23] Although the underlying mechanism of the gender-specific discrepancies in smoking-related BP reductions remains unknown, it might be due to social, physiological, and behavioral factors associated with smoking [24,25]. Nevertheless, considering the limited number of female smokers in the current study, it is possible that our finding of the greater association between smoking and BP among females is due to chance, and the result should be interpreted with caution. The mechanism and gender differences of this relation between smoking and BP need future investigations. 

However, Primatesta et al. [26] found that the SBP of regular smokers was higher than that of never smokers in men. The reason for the paradoxical association between smoking and BP levels is still unknown. Several studies reported smoking was related to reductions in body weight, while weight loss could cause decreased BP levels in smokers [27,28]. Additionally, a rebound phenomenon and adaptation process also may explain the reductions in BP levels among smokers [29]. Furthermore, smokers may not quit smoking since they are in good health [30]. Due to the complicated underlying mechanisms regarding the relationship between smoking and BP, further longitudinal data are needed to further explore the association between smoking and BP levels. Additionally, despite the association between smoking and BP being statistically significant in men, the magnitude of the effect was tiny. Therefore, the clinical impact of smoking on BP reductions, especially in males, should be further clarified. 

Additionally, the association between alcohol consumption and BP levels has been explored widely [21,31] A previous meta-analysis showed that drinking two or fewer drinks per day was not associated with a BP increase, while drinking more than two drinks per day was associated with increased BP [21], which was similar to the results among males in our study. Wakabayashi, I. [32] also suggested alcohol consumption was more prone to be associated with higher BP levels in men than in women. The gender-specific effect of regular alcohol consumption on BP levels may be caused by differences in alcohol consumption and metabolism patterns in men and women [33].

Smoking and drinking behaviors tend to occur together [13,14]. The proportion of regular smokers among regular drinkers was 68.23% in the present study. Alcohol consumption affected the relationship between smoking and BP levels, while the relationship between alcohol consumption and BP did vary by smoking status [23]. However, their combined effects on BP levels remain controversial. A previous study showed smoking modified the effect of alcohol consumption on SBP and DBP increases by 2–8 mm Hg in men and by 1–14 mm Hg in women [34]. We observed a significant interaction between regular smoking and alcohol consumption on BP increases in the current analysis, which showed the interaction between regular smoking and alcohol consumption caused SBP and DBP increases by 0.88–2.38 mm Hg in men and 1.29–5.21 mm Hg in women. The underlying mechanism may be that the neurochemical action of nicotine and alcohol could mutually reinforce, where neuronal nicotinic acetylcholine receptors play an important role in the effects of alcohol consumption behaviors [35,36] Considering the quantitative interaction analysis, we observed that the interaction between heavy smoking and drinking was broadly significantly associated with elevated BP levels. These results suggested individuals who smoked and drank alcohol regularly might be at greater risk of higher SBP and DBP when compared with subjects who only smoked, which revealed it is important to avoid heavy drinking among those who smoke regularly due to the increase of BP levels.

### Strengths and Limitations of the Study

Our study is a baseline data analysis of the CKB study, a national cohort study including participants geographically spread across urban and rural China. The study has a large sample size and a wealth of information on tobacco smoking and alcohol consumption, enabling us to conduct comprehensive stratified analyses. We conducted the analyses after standard data quality control and with careful adjustment for potential confounders. Nevertheless, this population-based study has some limitations. First, we only could observe a relationship between smoking and BP levels while failing to establish a causal association since the baseline survey of the CKB study was a cross-sectional study. Therefore, prospective longitudinal studies are needed to further confirm these results. Additionally, it is well-recognized that dietary factors are associated with the risk of hypertension, whereas smokers and non-smokers are reported to have different dietary habits [37]. Previous studies have shown that smokers may have a higher energy intake, consume less fruit and vegetables, and adopt poorer dietary habits when compared with non-smokers [38,39]. Furthermore, smoking might influence the preference for salt [40,41]. Thus, diet may confound the BP–smoking association. However, the detailed information on nutrient intake is not available in the CKB study. Further studies are warranted to explore the role of dietary factors in the relationship between smoking and blood pressure levels. 

## 5. Conclusions

The current study reveals a negative association between smoking and BP levels, and elucidates the potential effect modifier of alcohol consumption. Although the effect of interaction between regular smoking and alcohol consumption on BP increases was modest, the population-attributable risk of high BP associated with these risk factors may potentially be substantial considering the high prevalence of hypertension. Thus, public health interventions to reduce the burden of smoking and alcohol consumption remain an effective strategy in addition to antihypertensive treatment for reducing BP.

## Figures and Tables

**Table 1:** Characteristics of the study populations by smoking status.

	Non-Smokers (*n* = 294,715)	Ex-Smokers (*n* = 17,577)	Occasional Smokers (*n* = 27,075)	Regular Smokers (*n* = 67,914)	*p*
Age (years)	50.34 ± 10.39	54.82 ± 10.92	50.37 ± 10.94	51.00 ± 10.24	<0.001
30–49	145,371 (49.32)	5827 (33.15)	13,636 (50.36)	56,142 (46.61)	
50–69	134,612 (45.68)	9746 (55.45)	11,794 (43.56)	58,260 (48.37)	
70–79	14,732 (5.00)	2004 (11.40)	1645 (6.08)	6046 (5.02)	
Sex					<0.001
Male	27,345 (9.28)	16,371 (93.14)	21,980 (81.18)	114,540 (95.09)	
Female	267,370 (90.72)	1206 (6.86)	5095 (18.82)	5908 (4.91)	
Education					<0.001
Illiterate	68,607 (23.28)	1762 (10.02)	2286 (8.44)	12,617 (10.48)	
Primary school	89,741 (30.45)	5701 (32.43)	7871 (29.07)	42,508 (35.29)	
Middle school	77,510 (26.30)	5546 (31.55)	8630 (31.87)	40,420 (33.56)	
High school	42,679 (14.48)	3056 (17.39)	5456 (20.15)	19,151 (15.90)	
College and above	16,178 (5.49)	1512 (8.60)	2832 (10.46)	5752 (4.78)	
Alcohol consumption					<0.001
Non-drinkers	181,790 (61.68)	3144 (17.89)	4783 (17.67)	22,727 (18.87)	
Ex-drinkers	2802 (0.95)	1755 (9.98)	1460 (5.39)	8493 (7.05)	
Occasional drinkers	100,743 (34.18)	6427 (36.56)	14,888 (54.99)	42,889 (35.61)	
Regular drinkers	9380 (3.18)	6251 (35.56)	5944 (21.95)	46,339 (38.47)	
MET-hours/wk	20.92 ± 13.01	20.65 ± 15.10	22.62 ± 14.80	23.57 ± 15.21	<0.001
BMI (kg/m^2^)	23.78 ± 3.36	24.33 ± 3.21	23.86 ± 3.19	23.03 ± 3.19	<0.001
BMI < 18.5	11,625 (3.94)	430 (2.45)	843 (3.11)	6310 (5.24)	
18.5 ≤ BMI < 23.9	150,904 (51.20)	7779 (44.26)	13,531 (49.98)	70,743 (58.73)	
24.0 ≤ BMI < 27.9	99,415 (33.73)	7115 (40.48)	9894 (36.54)	34,590 (28.72)	
BMI ≥ 28.0	32,771 (11.12)	2253 (12.82)	2807 (10.37)	8805 (7.31)	
Hypertension	95,935 (32.55)	7633 (43.43)	8945 (33.04)	39,617 (32.89)	<0.001
SBP (mm Hg)	131.13 ± 23.28	137.13 ± 21.45	132.01 ± 20.68	132.21 ± 20.86	
DBP (mm Hg)	77.96 ± 11.87	81.51 ± 12.02	79.26 ± 11.79	79.26 ± 11.99	

Data are expressed as mean ± SD or *n* (proportion %). MET-hours/wk: metabolic equivalent hours per wk; BMI: body mass index; SBP: systolic blood pressure; DBP: diastolic blood pressure. *p* values were calculated using the one-way analysis of variance for continuous variables and a chi-square test for categorical variables.

**Table 2:** Multivariable ^a^ linear regression coefficients (95% confidence interval) for systolic and diastolic blood pressure levels according to baseline smoking status.

	Men (*n* = 180,236)		Women (*n* = 279,579)	
	SBP	DBP	SBP	DBP
Non-smoking (*n* = 294,715)	-	-	-	-
Ex-smoking (*n* = 17,577)	0.11 (−0.26 to 0.48)	0.23 * (0.01 to 0.45)	−5.93 *** (−7.09 to −4.77)	−2.41 *** (−3.05 to −1.77)
Occasional smoking (*n* = 27,075)	−0.08 (−0.42 to 0.26)	−0.20 (−0.40 to 0.01)	−3.11 *** (−3.68 to −2.54)	−1.01 *** (−1.32 to −0.69)
Regular smoking (*n* = 120,448)	−0.57 *** (−0.83 to −0.31)	−0.35 *** (−0.50 to −0.19)	−6.00 *** (−6.54 to −5.47)	−2.19 *** (−2.48 to −1.89)

^a^ Adjusted for age, body mass index, physical activity, educational level, and alcohol consumption. SBP: systolic blood pressure; DBP: diastolic blood pressure. * *p* < 0.05; *** *p* < 0.001.

**Table 3:** Multivariable ^a^ linear regression coefficients (95% confidence interval) for systolic and diastolic blood pressure levels according to smoking amounts ^b.^

	Men (*n* = 141,885)		Women (*n* = 273,278)	
	SBP	DBP	SBP	DBP
Non-smoking (*n* = 294,715)	-	-	-	-
Light smoking (*n* = 29,498)	0.18 (−0.15 to 0.51)	−0.09 (−0.29 to 0.10)	−5.55 *** (−6.25 to −4.84)	−2.04 *** (−2.43 to −1.66)
Medium smoking (*n* = 31,058)	−0.44 ** (−0.77 to −0.12)	−0.22 * (−0.42 to −0.03)	−6.63 *** (−7.61 to −5.66)	−2.32 *** (−2.86 to −1.79)
Heavy smoking (*n* = 59,892)	−1.07 *** (−1.35 to −0.78)	−0.50 *** (−0.67 to −0.33)	−6.47 *** (−7.83 to −5.10)	−2.52 *** (−3.27 to −1.77)
Pack-years/1000 (*n* = 120,448)	−0.04 *** (−0.04 to −0.03)	−0.01 *** (−0.01 to −0.01)	−0.06 ** (−0.11 to −0.02)	−0.04 ** (−0.06 to −0.01)

^a^ Adjusted for age, body mass index, physical activity, educational level, and alcohol consumption. ^b^ Only nonsmokers and regular smokers were included. SBP: systolic blood pressure; DBP: diastolic blood pressure. * *p* < 0.05; ** *p* < 0.01; *** *p* < 0.001.

**Table 4:** Multivariable ^a^ linear regression coefficients (95% confidence interval) for systolic and diastolic blood pressure levels according to smoking and alcohol consumption status in men.

	Non-Drinking (*n* = 35,208)	Ex-Drinking (*n* = 12,346)	Occasional Drinking (*n* = 70,516)	Regular Drinking (*n* = 62,166)
Systolic blood pressure				
Non-smoking (*n* = 27,345)	-	0.66 (−0.48 to 1.80)	−2.42 *** (−2.94 to −1.90)	−0.06 (−0.71 to 0.59)
Ex-smoking (*n* = 16,371)	0.01 (−0.81 to 0.83)	−1.07 (−2.70 to 0.56)	0.59 (−0.42 to 1.60)	0.70 (−0.38 to 1.78)
Occasional smoking (*n* = 21,980)	0.07 (−0.68 to 0.83)	−1.54 (−3.23 to 0.14)	0.23 (−0.66 to 1.12)	−0.11 (−1.15 to 0.94)
Regular smoking (*n* = 114,540)	−1.64 *** (−2.12 to −1.17)	0.93 (−0.30 to 2.17)	0.98 ** (0.37 to 1.59)	2.38 *** (1.66 to 3.10)
Diastolic blood pressure				
Non-smoking (*n* = 27,345)	-	1.10 ** (0.42 to 1.77)	−0.92 *** (−1.23 to −0.61)	1.45 *** (1.06 to 1.84)
Ex-smoking (*n* = 16,371)	0.07 (−0.42 to 0.55)	−0.26 (−1.22 to 0.71)	0.30 (−0.30 to 0.90)	0.50 (−0.14 to 1.14)
Occasional smoking (*n* = 21,980)	0.02 (−0.43 to 0.46)	−0.36 (−1.37 to 0.64)	−0.18 (−0.71 to 0.35)	−0.22 (−0.84 to 0.40)
Regular smoking (*n* = 114,540)	−0.62 *** (−0.90 to −0.33)	−0.01 (−0.75 to 0.72)	0.11 (−0.26 to 0.47)	0.89 *** (0.46 to 1.31)

^a^ Adjusted for age, body mass index, physical activity, and educational level. ** *p* < 0.01; *** *p* < 0.001.

**Table 5:** Multivariable ^a^ linear regression coefficients (95% confidence interval) for systolic and diastolic blood pressure levels according to smoking and alcohol consumption status in women.

	Non-Drinking (*n* = 177,236)	Ex-Drinking (*n*= 2164)	Occasional Drinking (*n*= 94,431)	Regular Drinking (*n*= 5748)
Systolic blood pressure				
Non-smoking (*n* = 267,370)	-	−1.07 * (−2.08 to −0.07)	−2.40 *** (−2.57 to −2.23)	−3.42 *** (−4.05 to −2.79)
Ex-smoking (*n* = 1206)	−6.08 *** (−7.88 to −4.27)	−1.63 (−6.09 to 2.83)	0.12 (−2.44 to 2.67)	3.15 (−0.94 to 7.24)
Occasional smoking (*n* = 5095)	−3.57 *** (−4.62 to −2.52)	1.14 (−1.84 to 4.12)	0.49 (−0.81 to 1.79)	1.93 (−0.13 to 4.00)
Regular smoking (*n* = 5908)	−7.46 *** (−8.27 to −6.66)	3.30 * (0.38 to 6.23)	1.75 ** (0.58 to 2.91)	5.21 *** (3.51 to 6.90)
Diastolic blood pressure				
Non-smoking (*n* = 267,370)	-	0.15 (−0.40 to 0.71)	−1.16 *** (−1.26 to −1.07)	−0.55 ** (−0.89 to −0.21)
Ex-smoking (*n* = 1206)	−3.01 *** (−4.00 to −2.02)	−1.16 (−3.61 to 1.28)	1.23 (−0.17 to 2.64)	2.23 (−0.01 to 4.48)
Occasional smoking (*n* = 5095)	−1.45 *** (−2.03 to −0.87)	0.46 (−1.18 to 2.10)	0.73 * (0.02 to 1.44)	0.50 (−0.63 to 1.64)
Regular smoking (*n* = 5908)	−3.10 *** (−3.55 to −2.66)	1.87 * (0.27 to 3.48)	1.29 *** (0.65 to 1.93)	2.62 *** (1.69 to 3.55)

^a^ Adjusted for age, body mass index, physical activity, and educational level. * *p* < 0.05; ** *p* < 0.01; *** *p* < 0.001.

**Table 6:** Multivariable ^a^ linear regression coefficients (95% confidence interval) for systolic and diastolic blood pressure levels among male participants ^b^ according to different amounts of smoking and alcohol consumption.

	Non-Drinking (*n* = 29,233)	Light Drinking (*n* = 17,591)	Medium Drinking (*n* = 23,996)	Heavy Drinking (*n* = 9082)
Systolic blood pressure				
Non-smoking (*n* = 14,202)	-	−2.26 *** (−3.11 to −1.41)	1.74 *** (0.80 to 2.67)	3.96 *** (2.17 to 5.76)
Light smoking (*n* = 13,596)	1.01 ** (0.34 to 1.68)	−0.68 (−1.87 to 0.51)	−1.70 ** (−2.93 to −0.47)	−0.68 (−2.89 to 1.53)
Medium smoking (*n* = 16,623)	−1.40 *** (−2.10 to −0.70)	2.72 *** (1.55 to 3.89)	0.85 (−0.34 to 2.05)	1.96 (−0.12 to 4.05)
Heavy smoking (*n* = 35,481)	−2.54 *** (−3.09 to −1.98)	3.06 *** (2.04 to 4.09)	1.99 *** (0.93 to 3.05)	2.84 ** (0.94 to 4.74)
Diastolic blood pressure				
Non-smoking (*n* = 14,202)	-	0.11 (−0.39 to 0.61)	2.41 *** (1.86 to 2.96)	4.16 *** (3.10 to 5.22)
Light smoking (*n* = 13,596)	0.63 ** (0.24 to 1.03)	−0.86 * (−1.56 to −0.15)	−1.23 ** (−1.96 to −0.51)	−1.06 (−2.36 to 0.24)
Medium smoking (*n* = 16,623)	−0.53 * (−0.94 to −0.12)	1.11 *^*^ (0.42 to 1.80)	0.32 (−0.38 to 1.03)	0.59 (−0.63 to 1.82)
Heavy smoking (*n* = 35,481)	−1.02 *** (−1.35 to −0.69)	0.79 * (0.18 to 1.39)	0.66 * (0.04 to 1.29)	0.87 (−0.25 to 1.99)

^a^ Adjusted for age, body mass index, physical activity, and educational level. ^b^ Only male non-smokers, non-drinkers, regular smokers, and regular drinkers were included in the analysis. * *p* < 0.05; ** *p* < 0.01; *** *p* < 0.001.

## Supplementary Materials

The following are available online at https://www.mdpi.com/1660-4601/17/8/2824/s1, Table S1: Characteristics of the study populations by alcohol consumption status., Table S2: Multivariable a linear regression coefficients (95% confidence interval) for systolic and diastolic blood pressure level according to baseline alcohol consumption status, Table S3: Blood pressure levels according to smoking and alcohol consumption status in men, Table S4: Blood pressure levels according to smoking and alcohol consumption status in women.
