# Supplementary material for: The Paradox Association between Smoking and Blood Pressure among Half Million Chinese People
Source: Int J Environ Res Public Health. 2020 Apr 20;17(8):2824. doi: 10.3390/ijerph17082824 (PMC7215755; doi:10.3390/ijerph17082824)
Supplement: Supplementary file 1 [file ijerph-17-02824-s001.pdf]

Supplementary

Table S1. Characteristics of the study populations by alcohol consumption status.

	**Non-drinkers (n = 212,444)**	**Ex-drinkers (n = 14,510)**	**Occasional drinkers (n = 164,947)**	**Regular drinkers (n = 67,914)**	p
Age (years)	51.63 ± 10.68	54.88 ± 10.18	49.15 ± 10.06	50.52 ± 10.02	<0.001
30–49	93,420 (43.97)	4,392 (30.27)	90,021 (54.58)	33,143 (48.8)	
50–69	105,463 (49.64)	8,862 (61.08)	68,419 (41.48)	31,668 (46.63)	
70–79	13,561 (6.38)	1,256 (8.66)	6,507 (3.94)	3,103 (4.57)	
Sex					<0.001
Male	35,208 (16.57)	12,346 (85.09)	70,516 (42.75)	62,166 (91.54)	
Female	177,236 (83.43)	2,164 (14.91)	94,431 (57.25)	5,748 (8.46)	
Education					<0.001
Illiterate	61,396 (28.9)	1,635 (11.27)	16,452 (9.97)	5,789 (8.52)	
Primary school	72,897 (34.31)	5,994 (41.31)	45,416 (27.53)	21,514 (31.68)	
Middle school	50,902 (23.96)	4,060 (27.98)	54,267 (32.9)	22,877 (33.69)	
High school	21,682 (10.21)	1,985 (13.68)	34,292 (20.79)	12,383 (18.23)	
College and above	5,567 (2.62)	836 (5.76)	14,520 (8.8)	5,351 (7.88)	
Smoking					<0.001
Non-smokers	181,790 (85.57)	2,802 (19.31)	100,743 (61.08)	9,380 (13.81)	
Ex-smokers	3,144 (1.48)	1,755 (12.10)	6,427 (3.90)	6,251 (9.20)	
Occasional smokers	4,783 (2.25)	1,460 (10.06)	14,888 (9.03)	5,944 (8.75)	
Regular smokers	22,727 (10.70)	8,493 (58.53)	42,889 (26.00)	46,339 (68.23)	
MET-hours/day	21.63 ± 13.68	19.68 ± 14.29	21.37 ± 13.65	23.19 ± 14.72	<0.001
BMI (kg/m^2)	23.46 ± 3.39	23.69 ± 3.37	23.77 ± 3.28	23.64 ± 3.19	<0.001
BMI < 18.5	10,979 (5.17)	661 (4.56)	5,306 (3.22)	2,262 (3.33)	
18.5 ≤ BMI < 23.9	113,928 (53.63)	7,251 (49.97)	85,793 (52.01)	35,985 (52.99)	
24.0 ≤ BMI < 27.9	66,626 (31.36)	5,074 (34.97)	56,186 (34.06)	23,128 (34.05)	
BMI ≥ 28.0	20,911 (9.84)	1,524 (10.5)	17,662 (10.71)	6,539 (9.63)	
Hypertension	75,502 (35.54)	6,083 (41.92)	45,146 (27.37)	25,399 (37.40)	<0.001
SBP (mm Hg)	132.99 ± 23.88	136.46 ± 23.3	128.58 ± 20.84	134.19 ± 20.69	
DBP (mm Hg)	78.4 ± 12.07	80.7 ± 12.64	77.33 ± 11.43	81.27 ± 12.03	

Data are expressed as mean ± SD or n (proportion %). MET-hours/wk: metabolic equivalent hours per wk; BMI: body mass index; SBP: systolic blood pressure; DBP: diastolic blood pressure. p values were calculated using the one-way analysis of variance for continuous variables and chi-square test for categorical variables.

Table S2. Multivariable [a] linear regression coefficients (95% confidence interval) for systolic and diastolic blood pressure level according to baseline alcohol consumption status.

	**Men (n = 180,236)**		**Women (n = 279,579)**	
	**SBP**	**DBP**	**SBP**	**DBP**
Non-drinking (reference)	-	-	-	-
Ex-drinking	0.88 *** (0.49 to 1.28)	0.98 *** (0.75 to 1.22)	-0.86 (-1.73 to 0.01)	0.20 (-0.28 to 0.67)
Occasional drinking	-1.75 *** (-2.00 to -1.50)	-0.85 *** (-0.99 to -0.70)	-2.36 *** (-2.53 to -2.20)	-1.12 *** (-1.21 to -1.03)
Regular drinking	1.57 *** (1.31 to 1.83)	2.08 *** (1.93 to 2.24)	-2.61 *** (-3.61 to -2.07)	-0.23 (-0.53 to 0.07)

[a] Adjusted for age, body mass index, physical activity, educational level, and smoking. SBP: systolic blood pressure; DBP: diastolic blood pressure. *$p < 0.05$; ** $p < 0.01$; *** $p < 0.001$

Table S3. Blood pressure levels according to smoking and alcohol consumption status in men.

	Non-Smoking	Ex-Smoking	Occasional Smoking	Regular Smoking
Systolic blood pressure				
Non-drinking	136.51 ± 22.89	138.75 ± 22.73	135.38 ± 22.49	132.59 ± 22.29
Ex-drinking	139.78 ± 23.69	139.73 ± 22.46	136.32 ± 22.47	135.89 ± 23.06
Occasional drinking	131.98 ± 19.80	134.82 ± 20.52	131.30 ± 19.23	129.33 ± 19.24
Regular drinking	135.83 ± 20.50	138.30 ± 20.88	133.90 ± 19.74	134.19 ± 20.45
Diastolic blood pressure				
Non-drinking	80.09 ± 12.33	80.82 ± 12.29	79.70 ± 12.26	78.26 ± 12.15
Ex-drinking	82.49 ± 12.66	82.74 ± 12.39	81.61 ± 12.85	80.40 ± 12.7
Occasional drinking	79.24 ± 11.36	80.27 ± 11.59	78.85 ± 11.42	77.73 ± 11.38
Regular drinking	82.39 ± 12.11	83.46 ± 11.91	81.75 ± 11.85	81.29 ± 12.02

Data are expressed as mean ± SD.

Table S4. Blood pressure levels according to smoking and alcohol consumption status in women.

	Non-Smoking	Ex-Smoking	Occasional Smoking	Regular Smoking
Systolic blood pressure				
Non-drinking	132.71 ± 24.11	138.99 ± 24.01	133.42 ± 24.38	132.14 ± 24.28
Ex-drinking	134.80 ± 24.72	133.00 ± 21.38	132.92 ± 25.74	133.84 ± 24.24
Occasional drinking	127.08 ± 21.64	133.00 ± 22.05	125.72 ± 20.87	127.86 ± 22.85
Regular drinking	128.31 ± 22.10	133.20 ± 24.93	127.40 ± 21.28	129.84 ± 22.25
Diastolic blood pressure				
Non-drinking	78.31 ± 12.04	77.94 ± 12.08	77.56 ± 12.18	75.95 ± 11.52
Ex-drinking	79.20 ± 12.03	75.91 ± 11.33	78.11 ± 12.94	77.98 ± 11.71
Occasional drinking	76.56 ± 11.37	77.39 ± 11.27	76.19 ± 10.89	75.62 ± 11.67
Regular drinking	77.45 ± 11.29	78.30 ± 12.58	76.77 ± 10.79	76.98 ± 11.77

Data are expressed as mean ± SD.
